# Self-Awareness and Stereotypes: Accurate Prediction of Implicit Gender Stereotyping

**DOI:** 10.1177/01461672221120703

**Published:** 2022-09-03

**Authors:** Zahra Rahmani Azad, Alexandra Goedderz, Adam Hahn

**Affiliations:** 1 University Hospital Tübingen, Germany; 2 University of Cologne, Germany; 3 University of Bath, UK

**Keywords:** implicit cognition, stereotypes, awareness, gender stereotypes, implicit attitudes, implicit association test

## Abstract

Research showing that people can predict the patterns of their implicit evaluations toward social groups has raised questions concerning how widely these findings extend to other domains, such as semantic implicit stereotyping. In a preregistered laboratory study, participants were asked to predict their scores on five implicit gender stereotyping Implicit Associations Tests (IATs). Within-subjects correlations between IAT score predictions and IAT scores showed high levels of accuracy. Although part of the IAT score patterns could be predicted from shared knowledge, own predictions significantly outperformed predictions of random others and normative patterns, suggesting self-awareness beyond reliance on shared knowledge. In line with dual-process models emphasizing that different information is captured by implicit as opposed to explicit measures, predictions explained correlations between implicit and traditional explicit stereotyping measures, and led to acknowledgment of bias. Discussion focuses on understanding conscious awareness of semantic automatic processes and conceptualizations of the cognitions underlying implicit measures.

One of the most prominent debates and points of contention in discussions around implicit measures revolves around whether they capture “unconscious” mental content.^1^ Early publications often listed unconsciousness as a defining characteristic of the outcomes of implicit measures (Greenwald & Banaji, 1995), and some researchers used the terms “implicit” and “unconscious” interchangeably (Lewis, 2018; Quillian, 2008). However, the assumption that implicit measures reflect unconscious mental content does not seem to be theoretically warranted (Gawronski et al., 2006; Hahn & Goedderz, 2020), and it has been challenged empirically. Specifically, in a series of studies, Hahn et al. (2014; see also Hahn & Gawronski, 2019) demonstrated high levels of congruence between predicted and actual patterns of implicit measures in the domain of ethnic and racial prejudice, supporting the idea that cognitions reflected on implicit measures can be available to conscious awareness. The purpose of this study is to extend these findings to the practically and theoretically important domain of gender stereotyping.

Gender stereotypes are prevalent in many societies and may result in gender discrimination (Bobbitt-Zeher, 2011). Women and men are regularly confronted with gender-specific expectations in their professional or private lives (Bian et al., 2017; Sullivan et al., 2018). Today, many people reject such gender-normative views, but there is a growing awareness in the popular media that even those who self-identify as feminist or egalitarian have spontaneous associations that echo the culturally learned stereotypes for women and men (Agarwal, 2020; Desmond-Harris, 2014; Devlin, 2018). This makes an investigation of awareness of spontaneous gender stereotyping an important area of study.

Another, theoretical, reason to extend Hahn et al.’s (2014) findings to the domain of gender stereotyping is that, in contrast to racial biases, gender stereotypes reflect primarily semantic connections between genders and certain attributes rather than mere evaluations of these social categories (Glick & Fiske, 1997; Ramos et al., 2018). That is, although stereotypes often contain valence (Kurdi et al., 2019; Phills et al., 2020), implicit stereotyping has been successfully dissociated from implicit evaluations (Amodio & Devine, 2006) such that the two constructs may be considered distinct but related concepts. Important for the present research, Hahn et al. (2014) found that their participants accurately predicted their implicit *evaluations* of ethnic minorities. The purpose of this study is to see whether people would be able to predict outcomes of semantic implicit measures as well.

Specifically, theoretical models describe two necessary conditions for any mental process to reach consciousness, namely, (a) the strength of the signal the process produces and (b) how much attention is directed to said signal (Hahn & Goedderz, 2020; based on work by Dehaene & Naccache, 2001; Hofmann & Wilson, 2010). Implicit measures such as the Implicit Associations Test (IAT; Greenwald et al., 1998) are assumed to capture spontaneously activated information from long-term memory. We delineate two potential processes through which these spontaneously activated memories could reach awareness. However, only one of these processes would translate from mere evaluations to stereotyping.

The first process involves listening to one’s spontaneous affective “gut response” in response to the target stimuli (Gawronski & Bodenhausen, 2006; Hahn et al., 2014; Hahn & Gawronski, 2019). That is, participants in Hahn et al.’s study (2014) were asked to look at pictures of different groups contrasted with each other on the same page (e.g., Black and White people) and asked to predict how positive or negative their reactions would be across five different IATs. Their accurate predictions may reflect a spontaneous affective response to these groups (Hahn & Goedderz, 2020; Ranganath et al., 2008).

In this study on gender stereotyping, participants were asked to predict how strongly pictures of the same groups are associated with different attributes (e.g., women with language and men with mathematics). Relying merely on one’s affective reactions toward the stimuli should yield limited information for this task. For instance, if a person felt more positive reactions toward stimuli of women than men or vice versa, they would still have to figure out how these positive reactions match onto five pairs of attributes differentially. The affective signal of these attributes, however, will vary across attribute pairs, and many stereotypic attributes may not produce a strong affective signal at all (Amodio & Devine, 2006).

To complicate matters further, people tend to have more positive reactions toward women (dubbed the “women-are-wonderful effect,” e.g., Nosek & Banaji, 2001; Richeson & Ambady, 2001; Rudman & Goodwin, 2004), but often ascribe more positive characteristics to men (e.g., “men are more competent than women,” Moss-Racusin et al. (2012); or “brilliance is male,” Bian et al., 2017). Hence, merely making a prediction on the basis of matching affective reactions would (a) sometimes be uninformative (whenever the attributes are more neutral in valence), (b) it would often not suffice to predict patterns of stereotyping (whenever the strength of the valence of the attributes varies from pair to pair), and (c) it would often lead to inaccurate predictions (whenever the more positive attribute is associated with the disliked group).^2^

Accordingly, if attention to affective reactions toward social groups is the main mechanism driving the accurate predictions of implicit outcomes, it would be harder to predict one’s implicit stereotyping scores. Consequently, prediction accuracy for implicit stereotyping measures should be lower than for implicit measures of prejudice.

A second potential strategy when asked to make such predictions is to judge the fluency with which an association comes to one’s mind (Unkelbach & Greifeneder, 2013). Is it easier to imagine a male or female mathematician? What gender comes to mind when one thinks of a stay-at-home parent? If assessment of fluency was the underlying process of predicting implicit measures, the prediction of implicit stereotyping measures would be at least as feasible as of implicit measures of prejudice. In addition, there is ample intergroup contact between men and women and stereotypical ascriptions might be quite salient. Intergroup contact between different ethnicities is often less frequent, especially in the context of socially homogeneous university campuses. Thus, if fluency judgments guided the predictions of implicit measures, the prediction accuracy for implicit gender stereotypes might potentially be even higher than for implicit racial prejudice.

In sum, in addition to addressing an important social phenomenon, resolving whether awareness is possible for spontaneous gender stereotyping can provide insights into the mechanisms underlying the process of reaching awareness of the spontaneous cognitions reflected on implicit measures.

To study these questions, we tried to find five commonly described gender stereotypes whose evaluative connotations would be both relatively neutral and balanced across genders (example of strongly valenced stereotypes: “Men are more criminal/smarter than women”). We selected five pairs that fulfilled our criteria: *mathematics* vs. *language*, *arts* vs. *sports*, *self-interest* vs. *other-interest*, *family* vs. *career*, and *rational* vs. *emotional*.

## The Present Research

The purpose of the study was to test whether people can show awareness of their spontaneous stereotypes. We introduced participants to the concept of automatic cognitions and asked them to predict their scores for five different gender stereotyping IATs, before they completed them. Our main outcome of interest was the within-subjects correlation between participants’ predictions and their actual IAT scores. Except for minor variations in the layout and order of the stimuli presentation (described in further detail in the method section), all participants followed the same procedure with identical material. We report all measures and exclusions. All data, hypotheses, and study materials, as well as a preregistration of the study, can be found at https://osf.io/5gmws/.

## Method

### Participants

We originally aimed at recruiting 140 to 160 participants at the University of Cologne, Germany, via direct approach, posters, and flyers in return for course credit or monetary compensation. However, our preregistered plans were foiled by the exacerbation of the COVID-19 pandemic, which led to a halt of all data collections in our laboratory in mid-March 2020. At this point, we had yielded a sample size of 90 participants. Given the within-subject design of our study, we decided that this sample size was large enough to test our hypotheses with adequate power. Of the 90 subjects who participated, four people indicated that they were non-native German speakers and were excluded in line with our pre-registration protocol. The remaining 86 participants were aged between 18 and 69 years (median age was 24 years, 73% female, 27% male), and 85 out of 86 participants were current university students.

### Design and Measures

Materials consisted of explicit endorsements of stereotypes, followed by explanatory texts about the concept of “implicit attitudes,” IAT score predictions, and IATs. They were the same for all participants except for two minor variations: the block order of the IATs and the layout of the prediction slides varied between participants (see below for details). This resulted in a 2 (IAT block order, IBO) × 2 (female pictures were presented above vs. below the male pictures on the prediction slide) design. As preregistered, we did not expect these minor differences in the layout and procedure to influence the results.

#### Explicit beliefs

To measure participants’ explicit endorsement of the gender stereotypes, they were asked to indicate how strongly they associate the 10 stereotype labels presented on the screen in pairs of two (family—career, rational—emotional, language—mathematics, self-interest—other-interest, arts—sports) with male or female gender. They moved sliders on 11-point scales (from 0—*very much with women* to 10—*very much with men*). Participants always rated the two stereotypical characteristics that were contrasted in the IATs and the IAT score predictions on the same page (e.g., “to what degree do you associate family-oriented . . .” and “. . . career-oriented with men or women” were rated on the same page). Both sliders could be moved independently, allowing for all possible combinations. Participants completed those ratings twice, once in the beginning of the study before the predictions and IATs, and once afterwards.

#### Explanatory texts

Participants were introduced to the concept of spontaneous reactions and learned that they do not have to align with their explicit beliefs. They also read a description about the IAT. The introductory texts were kept simple, did not use jargon or suggestive words like “stereotype,” and were illustrated by an example about cats and dogs and their characteristic traits of autonomy and loyalty, including both predictions for and completion of a very-short dog–cat autonomy-loyalty IAT.

#### Predictions of implicit stereotyping

**Figure 1.**
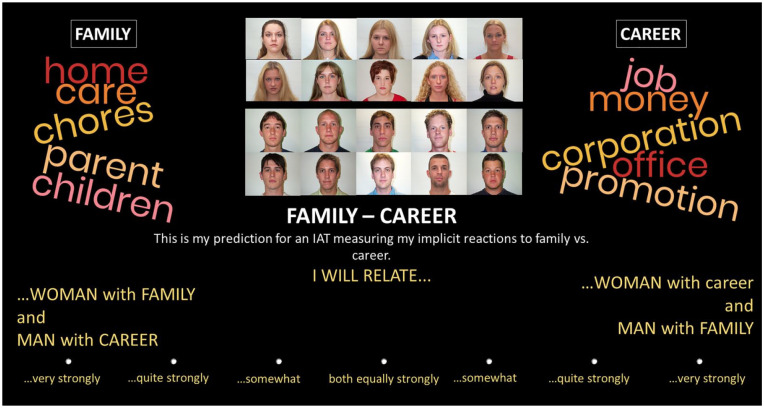
A sample prediction slide, translated to English from German. *Note.* Half of the participants saw pictures of women above pictures of men (as in this example), whereas the other half saw a reverse vertical ordering. Which concepts would show on which side horizontally was also varied between-participants (see text).

A sample prediction slide, translated to English, is shown in Figure 1. Participants were asked to predict how they would score on IATs that assessed associations of women and men with the five attribute pairs that were also compared on the explicit ratings. Participants saw all words that would be used on the IATs in word clouds to the right and left on top of the prediction slides, as well the 5 pictures each of men and women that would be used on the IATs in two rows in the top center of the screen. The exact words can be found in the appendix. We carefully chose unambiguous, nonnegated, evaluatively balanced, and grammatically gender-neutral words that are commonly used in German. Whether pictures of men or women were shown on top or below on the prediction slides was counterbalanced across participants, but was constant within each participant. Which of two contrasted concepts would show on the left or the right of the screen was also counterbalanced across participants. For half of the participants, the stereotypical response would be on one side in three of the predictions (language vs. mathematics, arts vs. sports, family vs. career) and on the other side for the other two (self- vs. other-interest, rational vs. emotional). For the other half of the participants, this was exactly reversed. We implemented these variations to increase vigilance and variance in participants’ predictions, to prevent participants from making the same constant prediction across the five IATs (leaving no variance in the predictions that could be used for analysis), and to make sure results were independent of these variations. Hence, there were four different versions of the design (pictures of men above vs. below pictures of men, and attributes on the right vs. the left). Participants completed one prediction for the dog-cat independent-loyal IAT and then completed a short version of the IAT. They completed the remaining predictions only after these explanations and examples.

#### Implicit association tests with gender stereotypes

Participants completed five word-picture stereotyping IATs in individually randomized orders. Each IAT compared the associative strength of the female or male gender (depicted by photos from Minear & Park, 2004, database) with words depicting the gender stereotypes described earlier. We used shortened IATs to avoid fatigue, with 20 iterations for the first practice block and 40 trials each for the three subsequent blocks (see Hahn et al., 2014).

Specifically, participants first completed one face-sorting task to familiarize themselves with the male and female photo stimuli for 20 trials. Half of the participants always sorted male pictures to the right and female pictures to the left, while the sides were reversed for the other half (first methodological variation: Sidedness). After that, this face-sorting block was not repeated, because the stimuli remained on the same sides in all five IATs for any one individual participant. The five IATs consisted of four blocks each. The first block was a 20-trial stereotype word-sorting practice block. In critical Block 2, participants assigned the words to the stereotype labels and the pictures to the gender labels simultaneously for 40 trials. Third, they completed a 40-trial reversed word-sorting block where the stereotype labels were horizontally flipped. In critical Block 4, participants again sorted both the word and picture stimuli over 40 trials, but the pairing was reversed compared with Block 2 and in line with training Block 3. The pictures of men and women were different for every IAT.

We obtained one *D*-score per IAT by subtracting the average reaction time of Block 2 from the average reaction time in Block 4, divided by their pooled standard deviation (Greenwald et al., 2003). There were two design conditions for the block order. For half of the participants (IAT block order, IBO, Condition 1), the language vs. mathematics, arts vs. sports, and family vs. career IATs were completed with the stereotype-consistent block first and the stereotype-inconsistent block second. Higher scores on these three IATs meant more stereotypical associations. The same participants completed the self- vs. other-interest and emotional vs. rational IATs such that the counter-stereotypical block was completed first and higher scores meant *less* stereotypical associations. For the other half of participants (IBO Condition 2), these block orders were exactly reversed. The prediction slides were completed in line with each participant’s block order condition, such that higher numbers would reflect more or less stereotypical associations in the same way as the IATs. To ease comparability across these methodological variations, we later reverse-scored all predictions and IAT scores in IBO Condition 2 to be similar in meaning to those of IBO Condition 1.

The reliability of the IATs, calculated by averaging the results of 600 split-half reliabilities (Cronbach’s α values) based on 600 random splits of the trials of each IAT, were satisfactory (Art vs. Sport: α = .72; Family vs. Career: α = .70; Language vs. Mathematics: α = .77; Other-Interest vs. Self-Interest: α = .62; Emotional vs. Rational: α = .77).

### Procedure

Participants were seated at individual cubicles with stationary computers. After signing informed consent, participants were randomly assigned to one of the four methodological conditions (IBO and sidedness). Participants started the study with the explicit ratings, completed in orders that were individually randomized for each participant. Next, they received instructions and completed a prediction task with a subsequent mini dog-cat-IAT for practice, and to understand how an IAT measures stereotyping. This was followed by the prediction procedure that ended on five predictions for five IATs, also completed in individually randomized orders. Participants then went on to complete the five IATs (in new randomized orders), before they repeated the explicit ratings (once again in new randomized orders). The instruction for the second explicit ratings said that we were interested in whether participants’ attitudes had changed after completion of the IATs. Finally, participants answered some demographic questions and had the option to provide feedback in free-response questions. Upon completion, participants were debriefed and reimbursed.

## Results

All analyses were conducted with R and R studio (R Core Team, 2020) and are available in the project repository (https://osf.io/5gmws/). Multilevel models were calculated using the software from the lme4 and lmerTest packages (Bates et al., 2015; Kuznetsova et al., 2017).

### Standardization of variables

The dependent variables and all predictor variables that were entered into the multilevel analyses were within-person standardized. Standardizing the variables beyond just centering them allowed us to look only at the accuracy with which participants predicted the patterns of their implicit stereotyping scores, independent of the variation in their scores and predictions.^3^ In addition, this standardization simplifies the interpretation of the regression coefficients: the regression weights of the models’ fixed effects of predictions correspond to the Pearson correlation coefficients between the IAT score predictions and the IAT scores per person. As a result of the standardization, all intercepts were zero and removed from the regression models (no-intercept models).

### IAT Scores

On average, participants were faster in the stereotype-congruent IAT-blocks than in the stereotype-incongruent blocks across all five stereotype domains. The IAT-D-scores, scored such that higher numbers meant stronger associations of the first attribute with men and the second with women, were: *D =* –0.39 for emotional vs. rational, *D =* 0.39 for sports vs. arts, *D* = 0.33 for career vs. family, *D* = 0.28 for mathematics vs. language, and *D* = –0.21 for other-interest vs. self-interest. According to common conventions, these implicit gender stereotyping scores can be labeled as slight to moderate.

### Awareness

To estimate the prediction accuracy for individual participants across the five different IATs, we regressed the person-standardized IAT-D-scores onto the predicted IAT scores in a multilevel model with all data points nested within each individual. The multilevel models included a random slope for each participant to account for Level 2 variance in participants’ accuracy. The results from the multilevel model can be seen in the first column of Table 1.

**Table 1.** Multilevel Regression Models Predicting IAT-D-Scores From IAT Score Predictions and Explicit Stereotyping.

Predictors	Prediction model estimates	Implicit–explicit model estimates	Simultaneous model estimates
Fixed effects			
IAT score predictions	0.63 (0.04)***		0.60 (0.07)***
Explicit ratings		0.52 (0.05)***	0.07 (0.07)
Random effects *SD*			
Residuals	0.69***	0.76***	0.64***
IAT score predictions	0.13		0.30**
Explicit ratings		0.18	0.41***
Observations			
No. of obs.	430	430	430
No. of groups: participants	86	86	86
Goodness of fit			
Marginal *R*^2^/Conditional *R*^2^	0.393/0.410	0.250/0.280	0.401/0.494
AIC	919.77	1,009.76	913.63
BIC	931.96	1,021.95	938.01
Log-likelihood	–456.88	–501.88	–450.82

*Note*. The dependent variables in all models are IAT-D-scores. All models are random-slopes no-intercept models estimated with the REML method. All variables are standardized for each individual participant, rendering all intercepts zero. This way, regression coefficients can be interpreted like Pearson correlation coefficients in one-predictor models, or part-correlations in simultaneous predictions, respectively. Standard errors are in parentheses. IAT = Implicit Associations Test; AIC = Akaike information criterion; BIC = Bayesian information criterion.

** 99% Confidence Interval does not include zero.

*** 99.9% Confidence Interval does not include zero.

As can be seen, the average prediction accuracy was significant and large, *b* = 0.63, 95% CI [0.56, 0.71], *t*(83.00) = 15.80, *p* < .001. As described before, the size of the regression weight corresponds to the average correlation coefficient between predicted and actual IAT scores. The standard deviation of the random slopes was small, τ = 0.13 and nonsignificant, 95% CI [0, 0.26]. This indicates that the best fitting slopes were similar across participants.

Because the distribution of the correlations per person is right-skewed, with many high correlation coefficients and few low or negative correlation coefficients, we also computed correlations manually for each participant and looked at the resulting distribution across participants. The mean of the distribution was equal to the regression weight of the multilevel model, and the median was *r* = 0.76.^4^ Fisher-*z*-transforming these values revealed a mean value of 0.96 which back-transforms into a correlation coefficient of .75, closely matching the median accuracy of .76.

Neither sidedness (of men vs. women in both the predictions and the IATs), nor IBO showed any significant impact on prediction accuracy. Predictors for IBO, sidedness, and their interaction, did not interact with prediction accuracy; all *F*s < 1, all *p*s > .27, all *R*² ~ 0.

### Explicit–Implicit Correspondence

Theoretical models claim that explicit and implicit measures capture the results of two different attitude retrieval processes, namely propositional and associative ones (Gawronski & Bodenhausen, 2006). If an attitude target activates another concept in the associative network but is propositionally rejected, implicit and explicit measures differ. A correspondence between IAT score predictions and actual IAT scores can only be interpreted as evidence for awareness if the results of implicit measures cannot be explained by explicit measures to the same degree. Hence, explicit measures need to be less predictive of IAT results than IAT score predictions to justify the conclusion that they capture different information, and that the spontaneous mental processes captured in IAT scores are often rejected for traditional explicit ratings. In contrast, the relationship between explicit measures and IAT scores *should* be explained entirely by IAT score predictions. As a result, a simultaneous regression where IAT scores are predicted by both IAT score predictions and explicit ratings should show no noticeable reduction in the predictive power of IAT score predictions, while the predictive power of explicit ratings should drop substantially.

To test these points, we ran the same multilevel analyses described previously, replacing IAT score predictions with explicit ratings as the main independent variable. This model revealed a significant correspondence between the patterns of participants’ explicit scores and their IAT-D-scores on a within-person-level, *b* = 0.52, 95% CI [0.43; 0.62], *t*(77.00) = 10.98, *p* < .001 (see Table 1, Column 2). However, when both predictors were included into the model, only the IAT-predictions, but not the explicit stereotype ratings, significantly explained variance in the dependent variable, IAT score predictions: *b* = 0.60, 95% CI [0.44; 0.74], *t*(46.06) = 8.82, *p* < .001, explicit ratings: *b* = 0.07, 95% CI [–0.07, 0.21], *t*(56.18) = 0.90, *p* = .37 (see Table 1, Column 3). Confirming predictions, the predictive power of IAT score predictions remained almost unchanged with the inclusion of explicit measures, whereas explicit measures were no longer predictive of IAT scores when IAT score predictions were included in the model (compare different columns of Table 1). Gauging principles of parsimony and goodness-of-fit, the model including IAT score predictions as the only predictor prevails over the other models (cf. Table 1).

### Do Participants Have Insight Into Their Own Unique Stereotyping Patterns, or Do They Draw Upon Shared Knowledge?

Which aspects of their IAT scores did participants predict? As outlined above, we programmed two of the gender stereotypes such that two of the IATs would always be completed in opposite order concerning stereotype-consistency (participants would complete the stereotype-consistent sorting first and the stereotype-inconsistent critical block second for three of the IATs, but reversed for the remaining two, or vice versa; see materials section), and this order was matched by the prediction slides such that higher numbers on the scales would match with higher numbers on the IATs. In addition to the advantages of this procedure in creating variance and increasing attention by switching sides as described above, however, this scoring allows participants to predict a significant portion of their scores based on just echoing the commonly known stereotypical assignment of traits, without any particular knowledge about the specific strength of these associations in their own minds. That is, by resorting to common cultural knowledge on gender roles, any participant should be able to correctly predict the sign of their IAT-D-scores as positive or negative (i.e., they will pair “men” with “rational” and “women” with “emotional” more readily than “men” with “emotional” and “women” with “rational”), and given these signs were reversed for two of the IATs compared with the other three, such a prediction would already look reasonably accurate in our models. However, if participants were able to gauge the idiosyncratic strength of these gender associations in their own minds, they should be able to predict variance beyond this particular contrast. We were interested to disentangle the relative contribution of these two aspects that both contribute to the prediction accuracy: direction (shared) and strength (idiosyncratic) of their automatic response. We conducted three analyses to investigate this point. They were not preregistered.

First, we ran another multilevel regression model including a binary variable “stereotype-consistent.” It coded those IATs where higher numbers indicated less stereotypical associations (emotional/rational and other-interest/self-interest) as –3 and the others as 2. This predictor captures the proportion of accuracy that can be explained by indicating the stereotype congruent direction of the implicit score. Thus, the remaining, partial regression weight for the predicted implicit stereotyping only captured if nuances beyond the mere direction of the stereotype were accurately predicted. The predictor for stereotype consistency was significant, *b* = 0.19, 95% CI [0.13, 0.25], *t*(8.09) = 6.36, *p* < .001. Importantly however, even when accounting for mere directionality of the implicit scores, participants’ IAT score forecasts remained a significant predictor, *b* = 0.21, 95% CI [0.09, 0.33], *t*(308.99) = 3.39, *p* < .001. This indicates that even when controlling for stereotype congruence, participants correctly predicted the relative strength of the respective gender stereotypes within their own patterns of scores. Hence, the strategy to simply reproduce existing cultural gender stereotypes cannot fully explain the high prediction accuracy that we found in our data.

As a second test of how much insight participants had into their own unique patterns beyond intersubjectively shared patterns, we looked at how well any participant’s predictions would explain any other participant’s IAT scores. Specifically, if all participants simply predicted the patterns of IAT scores based on the same knowledge of the same common stereotypes, this time including variance beyond a simple contrast, then any participant recruited from the same population should be as accurate in predicting any other participant’s IAT scores as they are accurate in predicting their own scores. Based on this reasoning first presented by Hahn et al. (2014), we expanded and refined an analysis the authors presented in their paper. Specifically, we ran a simulation in which every participant’s predictions now predicted the IAT results of a random other participant of the sample. This procedure was repeated for 1,000 iterations. For each of these 1,000 permutations, we ran a multilevel regression model and calculated the average regression weight of each iteration for the predicted IAT scores. The multilevel models were specified exactly like the Prediction Model (cf. Table 1, Column 1) that is described in detail above.

The average accuracy of all permuted datasets was *b* = 0.56. The highest estimate across the 1,000 permutations was *b* = 0.62, while the lowest was *b* = 0.50. This suggests that a large part of the predicted patterns could be explained by knowledge that was shared between participants and not idiosyncratic to any particular participant’s pattern of associations, even though the prediction accuracy remained lower in all 1,000 iterations than the prediction accuracy in the original dataset with participants’ own predictions, *b* = 0.63. Importantly, however, participants’ own predictions outperformed the random other predictions in all of 1,000 iterations.

Specifically, we ran another 1,000 iterations with random pairings, but this time, every participant’s IAT scores were regressed onto their own predictions and the predictions of a random other participant *simultaneously*. Results showed that participants’ own predictions outperformed the predictions of random others in 100% of the cases. That is, in all 1,000 cases of random permutations the regression, weight *b* was larger for the participant’s own predictions than for a random other person’s prediction, with the difference ranging from .002 to .441. On average, participants’ own predictions had a regression weight of *b* = 0.46, 99% CI = [0.40, 0.54], whereas the regression weight of the random other participant’s prediction over and above each participant’s own prediction was *b* = 0.25 on average, 99% CI = [0.14, 0.36]. Again, this demonstrates that while there is a shared pattern in every participant’s IAT scores that can be applied across participants, participants also have unique insights into the patterns of their own scores beyond this intersubjectively shared pattern, such that their own predictions always outperform the predictions made by random others on average, even in 1,000 pairing iterations.

Together, these three analyses show that although a notable proportion of the patterns of people’s IAT scores were shared across participants, echoing recent theorizing that automatically activated biases reflect cultural patterns (Payne et al., 2017), participants seemed to have unique insights into their own unique patterns of implicit gender stereotyping.

### Social Calibration

Previous studies sometimes concluded from low between-subjects correlations a lack of awareness of automatic attitudes (e.g., Nosek, 2005, 2007) As argued before (Hahn et al., 2014; Hahn & Goedderz, 2020), this level of analysis measures not only conscious accessibility of mental processes but rather the social calibration, that is, the intersubjective agreement of how to label the strength of an attitude. That is, in order for a between-subjects correlation between predicted and actual IAT scores to be high, the person with the strongest stereotype would have to know to make a more extreme prediction than the rest of the sample. This requires not only knowing one’s spontaneous stereotypes but also the ability to calibrate one’s strength in comparison to the remainder of the sample. In contrast, in order for within-subjects correlations to be high, participants need only know which of their stereotypes is stronger than which other stereotype (see Hahn et al., 2014; Hahn & Goedderz, 2020). To demonstrate the conceptual difference between social calibration and subjective awareness, we ran between-subjects analysis to assess the correlation between predicted IAT scores and actual IAT-D-scores. All predictions and IAT scores were standardized by IAT type. As a first overall analysis, we computed a multilevel-model regressing IAT-D-scores onto IAT score predictions by IAT type, controlling for IBO as a covariate. The overall between-subjects calibration was β = 0.24, 95% CI [ 0.14, 0.33], *t*(70.01) = 4.93, *p* < .001. All random effects were negligibly small, indicating that this average calibration accuracy did not vary meaningfully for the five IATs. The individual part-correlations per target pair can be seen in Table 2. These moderate-to-low relationships are in line with previous research documenting lower levels of social calibration than awareness for implicit bias measures (Hahn et al., 2014; Hahn & Goedderz, 2020).

**Table 2.** Social Calibration: Between-Subjects Correlations of Predicted IAT Results and Actual IAT Scores.

	Arts vs. sports	Family vs. career	Mathematics vs. language	Self-interest vs. other-interest	Rational vs. emotional	Average between-subject accuracy
β weight	0.17	0.24*	0.17	0.34**	0.21^†^	0.24***
95% CI	[–0.06, 0.39]	[0.02, 0.45]	[–0.05, 0.38]	[0.13, 0.54]	[0.00, 0.42]	[0.14, 0.33]

*Note.* Regression weights for participants’ IAT score predictions (standardized by IAT type). The dependent variable is the IAT-D-score. To control for block order effects, we also included a binary variable coding whether participants completed the IAT with the stereotype-consistent block or stereotype-inconsistent block first. These weights are not reported, none of them were significant, all *F*s < 1, all *p*s > .99. Due to standardization, the regression weights correspond to the part-correlations between predicted and actual IAT scores. Last column shows the average prediction accuracy (β-weight of predicted IAT score in the multilevel model with IAT type as random effect). IAT = Implicit Associations Test; CI = confidence interval.

† *p* = .051. *p < .05. **p < .01. ***p < .001.

### Adjustment of Explicit Stereotyping After Completing the IATs

As a last analysis, we conceptually replicated Hahn and Gawronski’s (2019) acknowledgment of bias effects in the domain of gender stereotyping. We ran a multilevel regression model with random slopes testing whether the patterns of explicit stereotypes changed to be more in line with implicit stereotyping scores after predicting and then completing the five gender stereotyping IATs. The IAT-D-scores as well as the explicit ratings before completing the IATs were entered as predictors for the post-prediction explicit stereotyping ratings. The results can be seen in Table 3.

**Table 3.** Multilevel Regression Model Measuring How Much Explicit Stereotyping Changed After Completion of the IATs as a Function of IAT-D-Scores.

DV: Explicit Stereotyping Post	
Predictors	Estimates
Fixed effects	
Explicit stereotyping pre	0.67 (0.04)***
IAT-D-score	0.20 (0.03)***
Random Effects *SD*	
Residuals	0.49***
IAT-D-score	0.09
Explicit stereotyping pre	0.13
Observations	
No. of observations	430
No. of groups: participants	89
Goodness of fit	
Marginal *R*^2^	0.661
AIC	637.64
BIC	662.03
Log-likelihood	–312.82

*Note*. *** 99.9% Confidence Interval for the effect does not include zero. All variables are standardized for each individual participant. Standard errors are in parentheses. AIC = Akaike information criterion; BIC = Bayesian information criterion.

Participants generally demonstrated stability in the explicit stereotyping they reported. This can be seen in that the post-procedure explicit stereotypes were strongly related to the same stereotypes reported before the prediction and IAT completion procedure, *b* = .67, 95% CI = [0.60; 0.74], *t*(69.69) = 18.74, *p* < .001. However, a significant slope of the IAT value showed that beyond this stability, participants also adjusted their explicit stereotypes significantly toward the cognitions reflected on the IATs, *b* = .20, 95% CI = [0.14; 0.27], *t*(89.21) = 6.15, *p* < .001. Participants did not receive any feedback on their performance in the IAT. Hence, these results replicate Hahn and Gawronski’s (2019) findings that predicting one’s IAT scores leads to alignment of explicit with implicit measures.

## Discussion

The purpose of the study was to examine whether participants can predict their unique patterns of implicit gender stereotyping IAT scores. Using a novel paradigm adapted from previous studies (Hahn et al., 2014; Hahn & Gawronski, 2019), we tested whether people could predict their IAT scores for gender stereotypes after they had received a brief introduction to the concept of automatic attitudes and indirect measurement instruments. Overall, the prediction accuracy for the IAT scores was high and all analyses unanimously indicated that predicting patterns of implicit stereotyping scores is possible with high levels of accuracy. Participants in the study were able to discern both the direction and the relative strength of their IAT scores for a set of five common gender stereotypes.

These findings are in line with models that assume that implicit and explicit measures of similar constructs are based on different information, such as the associative-propositional evaluations model (APE, Gawronski & Bodenhausen, 2006) or the Motivation and Opportunity as Determinants model (MODE, Fazio, 2007). In contrast to dual-systems approaches that describe implicit and explicit measures as capturing two distinct systems, one conscious and one unconscious (e.g., Greenwald & Banaji, 1995), models like the APE model assume that implicit and explicit measures capture different, but related content. While implicit measures are assumed to capture spontaneously activated mental content, explicit measures capture deliberative content that is salient at the moment a person is asked, and then further validated and probed for “truth” before it is endorsed. In line with this idea, the patterns of traditional explicit reports of stereotyping were less strongly correlated with IAT stereotyping scores in this study than IAT score predictions, and the relationship between traditional explicit measures and IAT scores were entirely explained by participants’ IAT score predictions. This supports the notion that spontaneously activated stereotypic associations are only one piece of information that factors into an explicit attitude report. Once this is controlled for, additional variance remains in explicit stereotyping scores that is unrelated to IAT scores. In addition, once spontaneous stereotypic associations were made salient to participants via the prediction procedure, the patterns of their explicit evaluations changed to become more in line with IAT scores. This also confirms the APE model’s assumption that explicit reports reflect information that is salient at the moment a person is asked (Hahn & Gawronski, 2019). In sum, the results of the present study are compatible with the APE model’s assumptions that implicit and explicit measures capture different but related information without assuming distinct systems.

Similarly, these findings are also compatible with Fazio’s (2007) MODE model. Specifically, according to the MODE model, implicit measures also capture spontaneous associations between concepts. If a person has the motivation and opportunity to override these spontaneous associations, their explicit reports will differ. Since participants continue to have the opportunity to override any spontaneous stereotypical associations on the IAT score predictions, the MODE model may be interpreted such that a prediction nullifies a person’s motivation to override their stereotypical response. In that sense, IAT score predictions might simply be more “honest” reports of stereotyping, akin to a “bogus pipeline” procedure (Jones & Sigall, 1971; Nier, 2005). While the present data are compatible with this interpretation, note that Hahn and Gawronski (2019) experimentally dissociated whether it was IAT score prediction or knowledge of measurement that led participants to discover their biases. Results strongly supported the former. IAT score prediction always led to alignment of explicit and implicit measures and acknowledgment of bias, whereas IAT completion alone (which includes full awareness of measurement of one’s biases) never led to any statistically noticeable changes on either an acknowledgment measure or explicit evaluations. Extrapolating from these findings to the current study would suggest that participants’ predictions were accurate because they made them pay attention to spontaneous reactions they would otherwise ignore or reject, and not because they made them “more honest” about their stereotypical thoughts because they knew they would be measured. Future research is needed to disentangle these different interpretations with respect to gender stereotyping.

As it stands, the current findings are compatible with a variety of dual-process models that assume that different, mutually interacting processes influence results on implicit and explicit measures, including the APE model (Gawronski & Bodenhausen, 2006) and the MODE model (Fazio, 2007), as well as other models that assume more than two interacting processes not discussed here (e.g., Cunningham et al., 2007; Van Bavel et al., 2012). They are also compatible with iterative multi-process models that assume a continuous interaction of reflective and automatic processes (e.g., Cunningham et al., 2007); as well as with single-process propositional models that assume that all mental links between concepts are propositional, while some information is activated more automatically than other information (e.g., Houwer, 2014; Hughes et al., 2011). However, they are less compatible with dual-systems models that assume two non-interacting, separate systems, especially those that assume that implicit measures capture “unconscious” representations (e.g., Greenwald & Banaji, 1995).

### Which Aspects of the IAT Score Patterns Did Participants Predict?

We interpret our results such that the IAT score predictions captured both shared normative patterns of gender stereotyping and unique knowledge of one’s own idiosyncratically learned gender associations. That is, on one hand, to increase vigilance in our participants and variance in IAT scores, the design of our study was such that knowledge of culturally normative patterns of stereotyping would already lead to accurate predictions. On the other hand, however, mere reproduction of normative stereotype patterns could not explain prediction accuracy in its entirety. Specifically, in our analyses, we consistently found that the variance in IAT scores that could be explained by participants’ predictions consisted of two portions. One portion was intersubjectively shared across participants. Thus, every participant’s predictions were informative about the patterns of IAT scores of a random other participant, and a binary predictor coding just the direction of the stereotypes explained a sizable proportion in IAT scores and prediction accuracy. At the same time, however, another portion was person-specific. Specifically, predictions of a random other participant were always less predictive of a person’s IAT scores than their own predictions in 1,000 random pairings of participants. Own predictions furthermore out-predicted both random other participants’ predictions in all of 1,000 pairing iterations, as well as a binary predictor for stereotype consistency.

Interestingly, while this last analysis confirmed that a portion of every participant’s accurate prediction was idiosyncratic, the portion that was shared was higher than we expected, and higher than what Hahn et al. (2014) found in their studies on intergroup prejudice. One explanation for this large amount of shared variance in stereotyping patterns might be restricted between-subjects variance. Our participants consisted of a homogeneous student sample with similar cultural backgrounds. Given that stereotypes are likely largely culturally learned, and demographic characteristics are known to predict results on indirect attitudinal measures (Nosek et al., 2007; Payne et al., 2017), this cultural homogeneity might have caused our sample to produce similar patterns of automatic responses on the IATs. Further research is needed to confirm if indeed random permutations will result in less-accurate other-predictions for more diverse samples. Taken together, the results suggest that the prediction patterns have both a culturally shared component and an individual component.

Another question concerns the processes that allowed participants to detect and quantify their own stereotyping patterns. Hahn et al. (2014) as well as Hahn and Gawronski (2019) argue that the cognitions reflected on IAT scores produce spontaneous affective reactions to which people must pay attention to notice their own biases (see also Goedderz & Hahn, 2022; Hahn & Goedderz, 2020; Phills et al. 2020; see also Kurdi et al., 2019) demonstrated that group-stereotyping scores and group evaluations are related, such that more negative evaluations predict more negative stereotyping, and more negative stereotyping predicts more negative evaluations. Hence, one may wonder whether people simply predicted their stereotyping scores based on how positive or negative their reactions toward women and men were. The design of the study with predictions of five different stereotyping IATs precluded this possibility.

Specifically, to predict an accurate pattern of stereotyping based on valence alone, a person would have to either always prefer the stereotypically female or the stereotypically male attribute. This may have been true for individual participants, but unlikely for enough participants to achieve a corrected mean accuracy coefficient of *r* = .75, considering different sources of measurement error in predictions and IAT scores, and considering the attribute pairs were chosen to be relatively neutral. That is, we assume that most people believe one should balance emotion and reason; that both arts and sports, and language and math skills are important; and that life is about balancing self and other-interest, and family and career aspirations. While individual participants may exist who prefer all of the attributes they associate with men (e.g., reason, sports, self-interest, careers, and math skills) over all of the attributes they associated with women (e.g., emotion, arts, other-interest, family, and language skills), and some of those participants may also show a preference for men over women, this is unlikely to be true for all participants. And even if it were, it could not explain why we found patterns of stereotyping scores beyond positive-negative. That is, rather than finding binary sets of equivalent scores where women were always associated with one (presumably positive) attribute to the exact same degree on all IATs, we found nuanced patterns of both predictions and IAT scores, and these predictions were highly accurate.

Group evaluations and stereotyping are related to the degree that the attributes in stereotypes are evaluative (Phills et al., 2020). In our study, too, participants with stronger preferences for women over men would certainly be more likely to stereotype women as emotional over rational to the degree that they consider being emotional more positive than being rational. The question, however, was whether they knew how much they associate women with emotion as opposed to other-orientation, family, language, and arts. The affective reaction to women alone wouldn’t allow for this prediction. To obtain accurate pattern predictions, participants had to predict the portion of variance that goes beyond comparing affective reactions to women with affective reactions to men. Hence, these affective reactions alone would not suffice to make these predictions with the degree of accuracy that we found.

This invites the question of how participants predicted their patterns of semantic associations. One possibility would be to construe the reaction upon which participants’ predictions are made more broadly as a general intuition, as the first thought that comes to mind. In this case, the process upon which people base their responses would not be a spontaneous affective reaction, but an observation of any spontaneous thought or association with gender, evaluative or not.

Another possibility is that participants might have derived their insights into their automatic reactions from the fluency that certain word-image pairs generated. Fluency creates a sense of positivity (Claypool et al., 2015; Wänke & Hansen, 2015). This could mean that stereotypic combinations would create more positive feelings than counter-stereotypic combinations. Alternatively, participants may have inferred from perceptions of fluency which combinations would be easier to complete on the IATs. Interestingly, this latter interpretation would indicate that affective reactions may have played a role in accurate semantic pairing after all, albeit in a less straight-forward way than in predictions of prejudice scores.

At present, both interpretations are compatible with the present data and the data presented by Hahn and colleagues. Participants might either (a) pay attention to the first thought that comes to mind when looking at the concepts or (b) pay attention to which combination “feels” better and more “fluent”; or both. Future research is needed to further clarify the role of affect in observation of one’s own spontaneous thoughts.

In sum, participants in this study were able to predict the patterns of their stereotyping scores, and this demonstrates high levels of awareness of the cognitions reflected on these IATs. That is, it is important to remember that participants readily applied these cultural patterns to themselves. This study did not ask participants what their culture would show or what other participants would show. Instead, we asked them to predict their own results on IATs. They may have drawn upon shared cultural knowledge and other sources to make these predictions. But that does not diminish the finding that they accurately predicted which biases they themselves would show on implicit tests, even when those went against their explicit beliefs. As such, this study provides empirical support for the idea that people can be aware not only of their automatic evaluative reactions but also of their automatic gender stereotypes.

### Limitations and Future Directions

This study was intended as a “proof-of-concept” to demonstrate that scores on semantic gender-IATs can be predicted accurately. However, as such, it poses the question of whether these findings generalize to other populations, other types of stereotypes (that are less culturally ingrained or have different valence), and other measures.

Our sample consists of a highly demographically homogeneous sample of university students. We see at least two aspects of this sample that might limit generalizations to other samples. First, young university students might be especially interested in the topic of gender stereotyping, and this may have led them to reflect on these stereotypes more before coming to our study. If this is true, other samples might show lower accuracy in predicting gender stereotyping. Second, the degree to which our participants showed similar patterns of gender stereotyping may be lower in other samples. Stereotypes, as well as cognitions reflected on implicit measures generally, are likely reflective of a person’s surrounding culture (Payne et al., 2017). Accordingly, the degree to which our sample showed overlap in stereotyping patterns might be smaller in more diverse samples. If this is true, the degree of unique insight into one’s own patterns may in fact be larger than our findings indicate. Importantly, however, both of these limitations concern minor aspects of our findings. That is, given that people around the world have ample contact with people of other genders, we believe the general finding that it is possible to predict the unique patterns of one’s own implicit gender stereotyping scores will likely generalize.

Concerning the measures we used, our attempt was to investigate whether people would be able to predict patterns of implicit measures that primarily assess semantic rather than primarily evaluative relationships between concepts. And in doing so, we tried to minimize the affective connotation of our semantic attributes. One may question whether we succeeded—the attribute pairs we chose may not be evaluatively neutral to all participants.

On one hand, we have explained above how we believe the within-subjects mapping of groups to five pairs of attributes require at least some processing of semantic information beyond listening to one’s affective reactions toward social groups. Hence, we believe that predicting patterns of stereotyping with one and the same group is nevertheless an extension over Hahn et al. (2014). On the other hand, if we believe that the attributes chosen in this study were not affectively neutral to all participants, this raises the question of how important the role of affect is even in the mapping of semantic relations in one’s mind. Future research is needed to test if truly valence-neutral stereotypes (e.g., those that are formed in a lab about fictitious social groups) are accessible to awareness as well.

Finally, we extended Hahn et al.’s (2014) findings to stereotyping by leaving many other aspects of the original authors’ paradigm—such as the IAT as the main instrument—similar. Many more and broader extensions to other measures, groups, and domains are necessary to gauge the generalizability of these findings. As it stands, the current findings show that accurate prediction of the patterns of one’s IAT scores is possible in the domain of mere evaluations and semantic stereotypes. Future studies will have to show how broadly this conclusion applies.

### Implications

Reframing spontaneously activated biases, stereotypes, and evaluations as accessible to consciousness has important ramifications both in theory and in practice. Theoretically, these findings contribute to a better understanding of implicit measures by adding another piece of evidence that refutes the idea that they capture inaccessible mental content. This is compatible with dual-process models of mental processes (e.g., Fazio, 2007; Gawronski & Bodenhausen, 2006) and may support more precise theorizing on different aspects of automaticity.

The finding that the IAT measures a construct available to conscious awareness has sometimes been used as evidence against the construct validity of implicit measures (e.g., Machery, 2022; Mitchell & Tetlock, 2017). In contrast to such reasoning, we believe that the fact that the IAT can correlate so highly with a self-report measure—given entirely different sources of measurement error—may be seen as support *for* its validity rather than the opposite (Kurdi et al., 2021). While we agree that there is a lot to be learned about the nature of the cognitions reflected on implicit measures and what they represent, the current studies show that what the IAT measures matches their perceptions of their own spontaneous thoughts. More research is needed to further elucidate the nature of such spontaneous cognitions.

In addition, our findings may be interpreted such that they question the usefulness of implicit measures. One may, after all, apparently just ask participants about their spontaneous reactions. Note, in this respect, that there are still a variety of aspects of people’s implicit stereotyping scores that predictions did not reveal. First, recall that participants were substantially less accurate in ranking the strength of their spontaneously activated biases in comparison to the other participants of their study than compared with their own other biases, as is illustrated in the social calibration analysis. The latter is in line with a battery of findings that people can only poorly rank the strength of their own automatic reactions among others’ automatic reactions (see Hahn & Goedderz, 2020; Hahn et al., 2014). Hence, even though our studies show that people may be able to observe their own gender biases, they seem to have limited knowledge on how much more or less stereotypical their thoughts are compared with the stereotypical thoughts of others.

Second, this study and the studies reported by Hahn et al. (2014) and Hahn and Gawronski (2019) involved confronting participants with a variety of concrete stimuli to reach awareness, and acknowledgment findings indicate that, to many participants, the discovery of their biases was new and unexpected. Together, these findings suggest that there are aspects about people’s spontaneous reactions toward social groups captured by the IAT that do remain outside of awareness, and future research is needed to elucidate factors that enable participants to make accurate predictions (Hahn & Goedderz, 2020).

Finally, the findings on awareness can also be useful in applied settings, for instance, to the design of anti-bias training or to tackle subtle sexist or racist reflexes more generally. In our last analysis, we looked at the acknowledgment effect of awareness and found that our data closely replicated findings by Hahn and Gawronski (2019). Taken together, these results can be interpreted as encouraging. It is much easier to tackle problems of which people can become aware, than to convince someone of harboring an “unconscious” bias they cannot perceive. As such, the current findings support the idea that people should be encouraged to observe their own biases instead of believing that their biases are guided by unconscious forces.

## Conclusion

Implicit evaluations or stereotyping are often framed as reflecting unconscious thought processes. The findings presented in this article contradict this notion and extend previous research on awareness of implicit evaluations to the important domain of primarily semantic gender stereotypes. They can thus contribute to gaining a better understanding about spontaneous mental processes in social cognition, and disentangle the concepts of automaticity and unconsciousness.

## Supplemental Material

sj-docx-1-psp-10.1177_01461672221120703 – Supplemental material for Self-Awareness and Stereotypes: Accurate Prediction of Implicit Gender Stereotyping Click here for additional data file. Supplemental material, sj-docx-1-psp-10.1177_01461672221120703 for Self-Awareness and Stereotypes: Accurate Prediction of Implicit Gender Stereotyping by Zahra Rahmani Azad, Alexandra Goedderz and Adam Hahn in Personality and Social Psychology Bulletin

## Appendix

Stimuli (translated from German) used in the gender stereotype IAT. Five stimulus words pertained to each stereotype label. Thus, every IAT had 10 different word stimuli presented in capital letters.

**Own-Interest:** ambition, individual, selfish, demand, assert

**Other-Interest:** compromise, community, together, help, concede

**Family:** children, home, care, household, parenting

**Career:** job, office, money, promotion, corporation

**Rational:** reason, rationality, factual, pragmatic, objective

**Emotional:** feeling, sensitive, warm, delicate, perceptive

**Language:** word, literature, read, write, poetry

**Mathematics:** number, algebra, equation, calculate, formula

**Arts:** theater, gallery, museum, concert, opera

**Sports**: soccer, stadium, league, match, tournament

Original stimuli used in the IATs in German:

**Eigenwohl**: Ehrgeiz, Individuum, egoistisch, fordern, durchsetzen

**Gemeinwohl**: Kompromiss, Gemeinschaft, zusammen, helfen, nachgeben

**Familie**: Kinder, Heim, Fürsorge, Haushalt, erziehen

**Karriere**: Unternehmen, Beruf, Geld, Beförderung, Büro

**Rational**: Verstand, Vernunft, sachlich, pragmatisch, objektiv

**Emotional**: Gefühl, sensibel, warm, empfindsam, feinfühlig

**Sprache**: Wort, Literatur, lesen, schreiben, Poesie

**Mathematik**: Zahl, Algebra, Gleichung, rechnen, Formeln

**Künste**: Theater, Galerie, Museum, Konzert, Oper

**Sport**: Fußball, Stadion, Bundesliga, Wettkampf, Turnier

## Notes

1 In line with Houwer (2014), we use the term “implicit” (for instance, implicit evaluation or implicit ascription) to describe the outcome of an indirect attitudinal measure that involves an automatic, that is, fast, spontaneous, or uncontrolled component. We do not categorize the latent attitude construct as implicit or explicit, but wish to help elucidate what these underlying constructs may be called with this research.

2 The interested reader might interject that implicit stereotyping and evaluation have been shown to be related (e.g., Kurdi et al., 2019; Phills et al., 2020). Note that the size of this correlation varies as a function of how evaluative the stereotypic attributes are (Phills et al., 2020). A remaining portion of the variance can be explained by nonvalent associations (e.g., Amodio & Devine, 2006; Kurdi et al., 2019). Hence, more positive evaluations of women will be correlated with positively stereotyping women (e.g., as socially capable). However, people may still stereotype women as more socially capable than physically strong due to prevailing cultural associations, even if both attributes are considered positive. It is this nonvalent portion of the variation in stereotyping scores and its predictability that we are interested in the current studies. We return to this point in the General Discussion.

3 The sizes of unstandardized slopes do not only vary as a function of the strength of the association, they also vary as a function of the variance in the scores. For example, a person who uses the prediction scale conservatively would end up showing larger unstandardized slopes than a person using the scale more liberally, even if the patterns they predicted were equally accurate within standardized scores. Therefore, we standardized all predictions and IAT scores within person (see Hahn et al., 2014; Hahn & Gawronski, 2019; Hahn & Goedderz, 2020).

4 A D’Agostino test for skewness (*z =* –10.04, *p* < .001) and an Anscombe-Glynn test for kurtosis (*z* = 5.06, *p* < .001) confirmed the deviation of the distribution of these correlations from normality. Fisher-*z*-transformation of all values led to a more normal distribution of scores (D’Agostino skewness test: *z =* –0.97, *p* = .33; Anscombe-Glynn kurtosis test: *z* = –0.03, *p* = .98).
