# Supplementary material for: Self-Awareness and Stereotypes: Accurate Prediction of Implicit Gender Stereotyping
Source: Pers Soc Psychol Bull. 2022 Sep 3;49(12):1695–708. doi: 10.1177/01461672221120703 (PMC10637100; doi:10.1177/01461672221120703)
Supplement: sj-docx-1-psp-10.1177_01461672221120703 – Supplemental material for Self-Awareness and Stereotypes: Accurate Prediction of Implicit Gender Stereotyping [file sj-docx-1-psp-10.1177_01461672221120703.docx]

Methodology File

1. **All Materials in English
   (Translated from German – see German original, including stimulus sets, below English version)**

**Slide 1**

HOW ARE MEN AND WOMEN?

In the first part of this study, we are asking you to rate how much you associate certain aspects of life with men, with women, with both genders equally, or with neither of the genders. On the next slide, you will see different terms and you should indicate whether you associate the term more with men or more with women. The response format will look like this:

I think, the term is more descriptive of

Men…………………………..…..same…………………….……..women

(11-pt range slider)

If you move the slider all the way to the left, this means you associate the term much more strongly with men than with women. If you move the slider all the way to the right, this means you associate the term much more strongly with women then with men. Moving the slider to the center would mean that you associate both men and women equally strongly or equally weakly with the term.

Continue by pressing the space bar.

**Slides 2 to 6 (presented in individually randomized orders):**

Do these terms fit better with men or with women?

I think “FAMILY” fits better with

Men…………………………..…..same…………………….……..women

(11-pt range slider)

I think “CAREER” fits better with

Men…………………………..…..same…………………….……..women

(11-pt range slider)

As above with

SELF-INTEREST and OTHER-INTEREST

ARTS and SPORTS

LANGUAGE and MATHEMATICS

RATIONAL and EMOTIONAL

____________________________________________________________________

**Slide 7 translated from German**

KNOWING YOUR IMPLICIT ATTITUDES

Please read the following instructions carefully. We want to make sure you understand the next task. The “continue” button is disabled for a while to ensure you will have had sufficient time to read these instructions.

Psychologists have long been interested in people's spontaneous reactions toward different people. That is, in addition to the things you say when you are asked about your attitudes, you may have spontaneous reactions toward people at first that you wouldn't always express. You might not even agree with those reactions, if you thought about them for a while.

For instance, if you imagine a Swabian person you might instantly think about the terms stinginess and ‘spaetzle’. However, you are well aware that Swabians are not necessarily stingy and can enjoy a variety of international dishes.

We call those attitudes that you would consider correct after some consideration and that you would openly report explicit attitudes, because you would report them explicitly when asked. We call the other kind of attitudes "implicit attitudes", because they show more implicitly in your spontaneous reactions.

____________________________________________________________________

**Slide 8 translated from German**

KNOWING YOUR IMPLICIT ATTITUDES

For this purpose, psychologists have developed a method called the "IMPLICIT ASSOCIATIONS TEST", or IAT for short. This test measures those implicit, i.e. spontaneous attitudes. In this study we are interested in whether you think that your implicit attitudes will differ from your explicit attitudes.

In the next part of the study, you will complete some IATs and we want to know whether you are able to predict your respective results. That is, we are curious if you know your own implicit, spontaneously activated attitudes towards men and women.

_____________________________________________________________

**Slide 9 translated from German**

HOW DOES THE IAT WORK?

But how can we measure attitudes that are only implicitly reflected in someone’s behavior?

Imagine you would have to sort a pile of cards onto two different stacks. These cards either show ‘spaetzle’ or hamburgers, or US-Americans or Swabians. You would probably find it easier to sort Americans and hamburger into one stack than to put cards with Americans or ‘spaetzle’ into one stack and hamburger and Swabians into the other stack.

The IAT makes use of this principle. To help you understand the IAT better, you will first complete a trial IAT with dogs and cats. But before you do this, we would already like to see if you can predict your reactions towards cats and dogs.

Click on the “continue” button, to make a prediction for the rehearsal IAT with cats and dogs.

___________________________________________________________________________

*The next slide was a prediction slide for the cat-dog-IAT. See layout in German version below.*

*The text says:*

Look at the pictures. All the pictures on the top belong to the category "DOG", all the pictures on the bottom belong to the category "CAT".

Now think about the descriptions "LOYAL and TRUSTFUL" vs. "AUTONOMOUS and OWNED" that are described next to it.

Listen to your gut feeling. What is your spontaneous reaction to these categories? Do you associate one of the descriptions more with cats and one more with dogs? Please click on the button that you think best reflects your implicit attitude toward DOG vs. CAT. If you take the choice in the middle, it means that you associate cats and dogs equally strongly or equally little with the characteristics LOYAL and AUTONOMOUS.

My prediction for an IAT that would measure my implicit responses to DOG vs. CAT is:

I associate ...

DOG with AUTONOMOUS

CAT with LOYAL

*The word categories for the Cat-Dog-IAT were* ***loyal*** *and* ***autonomous****.*

*The stimuli words that belonged to loyal were:*

*devoted, reliable, obedient, acquiescent, affectionate*

*The stimuli words that belonged to autonomous were:*

*free, headstrong, self-reliant, wayward, independent*

____________________________________________________________________________

**Transition slide before the Cat-Dog IAT started, translated from German:**

HOW ACCURATE WAS YOUR PREDICTION?

Next, you will complete an IAT, the implicit association test. The IAT measures your implicit attitudes towards cats and dogs. Were you able to predict them correctly?

The task is as follows. You will have to sort different words or pictures. With every trial, you will see a word or a picture in the middle of the screen which you are supposed to sort to a certain category.

It is important that you will sort the words and pictures as FAST as you can, but at the same time make as LITTLE MISTAKES as possible. If you are too slow or you make too many errors, your result cannot to evaluated.

_____________________________________________________________________

The task is to press one of two buttons when a word or a picture appears on the screen and thus assign it to a category on the left or right side.

The categories that each task is about will be shown at the top of the left and right corners of the screen, respectively.

Press the A key if something belongs to the left side.

Press the 5 key (on the right, on the numeric keypad) if something belongs to the right side.

The picture below shows exactly which keys we mean. It is best to keep your fingers on these keys during the task.

A red X appears every time you make a mistake - correct the mistake by pressing the other key.

(press spacebar to continue)

You will now start a trail IAT. You have just made a prediction about what your spontaneous reactions to cats or dogs will be.

This IAT measures your spontaneous reactions.

The two stimuli you have to distinguish between in this block are photos that belong to the categories CAT or DOG.

Press button A if a picture belongs to the category DOG.

Press key 5 if a photo belongs to the category CAT.

(press spacebar to continue).

Reminder:

Keep your fingers on the 'A' and '5' keys throughout the task.

If you press the wrong key, a red X will appear on the screen.

To correct it, press the other key.

*All instruction slides that follow are comparable in wording to the instructions on the two previous slides. The specific blocks are:*

*Participants now complete a short version of a dog-cat IAT with five blocks.*

*Block 1 has participants sort pictures of cats and dogs (see instructions above).*

*Block 2 has participants sort words pertaining to the concepts “loyal” and “autonomous.*

*In Block 3, participants sort pictures of cats and dogs and words simultaneously.*

*In Block 4, participants sort pictures to reversed sides.*

*In Block 5, participants again sort pictures and words at the same time, but the pairing is reversed.*

*See stimuli in prediction slide in the German version below*

____________________________________________________________________

**Transition slide 1 after the Cat-Dog IAT, translated from German:**

YOU HAVE COMPLETED YOUR TRIAL IAT.

How did feel during the IAT? Did you find that some pairings came more easily to you than others? The IAT measures if you it is easier for you to sort the categories autonomous and dogs together or if it is easier for you to sort the categories autonomous and cats together. From that, your spontaneous reactions are derived.

Please describe your reaction to the rehearsal IAT in your own words. What will your results show?

[Text box with free text input.]

__________________________________________________________

**Transition slide 2 after the Cat-Dog IAT, translated from German:**

Please consider your IMPLICIT ATTITUDES on women and men.

We will ask you about the following traits of men and women:

[order was randomized by line]

CAREER and FAMILY

EMOTIONAL and RATIONAL

MATHEMATICS and LANGUAGE

OTHER-INTEREST and SELF-INTEREST

SPORTS and ARTS

How do you expect your IAT (Implicit association test) results? Will the test show that you will associate some of the words spontaneously more strongly with women or with men? We are interested in your spontaneous reactions, so pay attention to your first impression when looking at the pictures and the words.

Do you know your spontaneous reaction, images and associations? Only you can tell us about that, so do your best.

After you have made your guesses, you will actually complete the IATs. How precise are you in predicting your results?

____________________________________________________________________

*Next, the five prediction slides for the gender stereotypes followed. They were as presented on Figure 1 of the paper. All prediction slides can be seen in the German version below. The concepts and words translate as:*

*Figure 1*
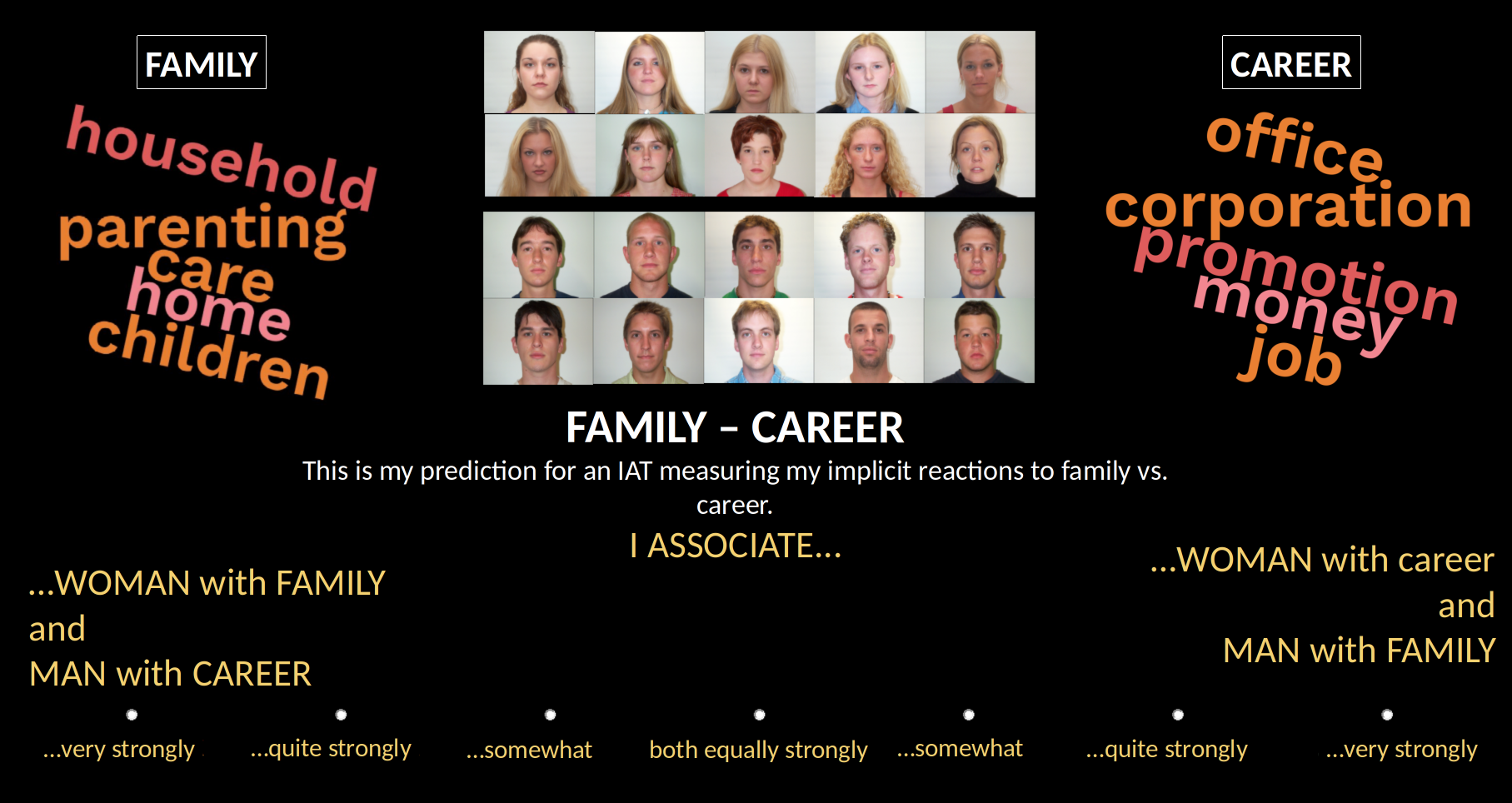
. A sample prediction slide, translated to English from German.

Stimuli (translated from German) used in the gender stereotype IAT. Five stimulus words pertained to each stereotype label, thus, every IAT had 10 different word stimuli.

**Own-Interest:** Ambition, Individual, Selfish, Demand, Assert

**Other-Interest:** compromise, community, together, help, concede

**Family:** children, home, care, household, parenting

**Career:** job, office, money, promotion, corporation

**Rational:** reason, rationality, factual, pragmatic, objective

**Emotional:** feeling, sensitive, warm, delicate, perceptive

**Language:** word, literature, read, write, poetry

**Mathematics:** number, algebra, equation, calculate, formula

**Arts:** theater, gallery, museum, concert, opera

**Sports**: soccer, stadium, league, match, tournament

You will now begin your IATs. The principle is the same as before when you sorted dog and cat pictures.

Reminder:

Please try to make as FEW MISTAKES and respond as FAST as possible.

Keep your fingers on the 'A' and '5' keys throughout the task.

***Participants now completed the five IATs, in individually randomized orders. Instructions were similar to the sample instructions shown above for the dog-cat IAT. See German version for the stimuli, and translation of word stimuli above.***

**Post IAT explicit attitudes slide, translated from German:**

HOW ARE MEN AND WOMEN?

Finally, we will ask you about your attitudes towards men and women with the same scales that you already used in the beginning of the study.

We are interested to see if your attitudes have changed or remained the same.

Continue by pressing the space bar.

*Wording of the post-experimental explicit ratings were the same as in the beginning.*

*Demographics*

1. Age ________
2. Gender: ( x) female (x) male (x) other
3. Migration Background:
4. I have no migration background
5. I was born in Germany, but one of my parents was not German at their birth
6. I was born in Germany, but both my parents were not born German
7. I was not born in Germany and moved here at one point in my life
8. [If C4 was selected]: At what age did you come to Germany? ____
9. What is your citizenship? (x) German (x) German and others (x) only others
10. [If E3 was selected]: What is your citizenship? _________________________
11. What language did you speak at home: (x) only German (x) German and other(s) (x) only other(s)
12. [If not G1] Please specify which other language(s) you spoke at home: ____________________________
13. What is your status at the University of Cologne? (x) Bachelor student (x) Master student (x) no student, but studied (x) no student, never studied
14. [if I1 or I2 was selected] What semester are you in? _______________
15. What is your field of study? (x) Psychology as major (x) another major but with psychology classes (x) another major without any psychology classes (x) I do not study and never have
16. What is your field of study? ___________________________________________________
17. Have you ever heard about the IAT or similar implicit measures? (x) yes (x) no
18. If yes, where? ________________________________________
19. Have you ever, before you participated in this study today, completed an IAT (here or elsewhere)? (x) yes (x) no
20. If yes, describe what you remember __________________________________
21. Do you believe you have ever participated in a similar study? (x) yes (x) no
22. Ich have already participated in a study, where I had to predict my IAT-results for ……
    1. Social and ethnical
    2. Pastries
    3. Professions
    4. Attitudes about genders
23. Can you describe your strategy when predicting your IAT scores? [large text box] ____________________________
24. When predicting your IAT results, how much attention did you pay to your intuition?

Not at all (o) (o) (o) (o) (o) (o) (o) very much

1. When predicting your IAT results, how much attention did you pay to what you learned culturally?

Not at all (o) (o) (o) (o) (o) (o) (o) very much

1. Do you have any feedback for us? [large text box] ____________________________
2. **Original German Materials**

**Slide 1**

WIE SIND MÄNNER UND FRAUEN?

Im ersten Teil der Studie wollen wir von dir wissen, wie sehr du bestimmte Aspekte des Lebens eher Männern, eher Frauen, beiden gleichermaßen, oder keinem der beiden Geschlechter zuordnen würdest. Du wirst gleich Begriffe gezeigt bekommen, und sollst angeben, ob du den Begriff eher mit Männern oder eher mit Frauen verbindest. Das Antwortformat sieht so aus:

Ich finde, der Begriff passt eher zu…

Männern………………..…………beiden gleich…………..………………….Frauen

(11-pt slider)

Wenn du den Slider ganz nach links bewegst, bedeutet das, dass du den Begriff viel stärker mit Männern als mit Frauen verbindest. Wenn du den Slider ganz nach rechts bewegst, bedeutet das umgekehrt, dass du den Begriff viel stärker mit Frauen als mit Männern verbindest. Wenn du den Slider in die Mitte bewegst, bedeutet das, dass du Männer und Frauen gleich stark oder wenig mit dem Begriff verbindest.

Weiter geht es mit der Leertaste.

**Slides 2 to 6 (presented in individually randomized orders):**

Passen die Begriffe eher zu Männern oder zu Frauen?

Ich finde „Familie“ passt besser zu

Männern………………..…………beiden gleich…………..………………….Frauen

(11-pt slider)

Ich finde „Karriere“ passt besser zu

Männern………………..…………beiden gleich…………..………………….Frauen

(11-pt slider)

*Wie oben mit*

*EIGENWOHL und GEMEINWOHL*

*KÜNSTE und SPORT*

*SPRACHE und MATHEMATIK*

*RATIONAL und EMOTIONAL*

**Slide 7**

DEINE IMPLIZITEN EINSTELLUNGEN KENNEN

Bitte lies die folgenden Instruktionen sorgfältig, um die nächste Aufgabe zu verstehen. Die 'Weiter'-Taste funktioniert erst nach einer Weile, um sicherzustellen, dass du genug Zeit zum Lesen hast.

In der Psychologie werden schon seit langem spontane Reaktionen untersucht, die Menschen gegenüber anderen Menschen zeigen. Zusätzlich zu deinen Einstellungen, die du äußerst, wenn du danach gefragt wirst, kannst du auch spontane Reaktionen haben, die du nicht unbedingt offen ausdrücken würdest und denen du vielleicht auch gar nicht zustimmen würdest, wenn du länger über sie nachdenkst.

Wenn du an eine schwäbische Person denkst, kommen dir vielleicht sofort die Begriffe Geiz und Spätzle in den Sinn. Du verbindest also spontan das Konzept 'schwäbisch' mit 'geizig' und einer regionalen Speise. Dennoch weißt du natürlich, dass Schwaben nicht immer geizig sind und auch eine Vielfalt von internationalen Gerichten genießen.

Einstellungen, die du nach einigem Nachdenken und Abwägen für richtig hältst oder du offen äußern würdest, nennen wir explizite Einstellungen, weil du sie explizit äußern würdest, wenn man dich fragt. Solche Einstellungen, die spontane Reaktionen sind, nennen wir implizit, weil sie sich eher implizit in deinen Reaktionen zeigen.

**Slide 8**

DEINE IMPLIZITEN EINSTELLUNGEN KENNEN

Aus diesem Grund wurde der IMPLIZITE ASSOZIATIONSTEST entwickelt, kurz IAT. Mit diesem Test sollen diese impliziten, also spontanen Einstellungen gemessen werden. In dieser Studie sind wir daran interessiert, ob du denkst, dass sich deine impliziten Einstellungen von deinen expliziten Einstellungen unterscheiden.

Gleich wirst du einige IATs durchführen und wir sind daran interessiert, ob du deine jeweiligen Ergebnisse vorhersagen kannst. Das heißt, wir sind neugierig, ob du deine eigenen impliziten, spontan aktivierten Einstellungen gegenüber Männern und Frauen kennst.

**Slide 9**

WIE FUNKTIONIERT DER IAT?

Aber wie kann man Einstellungen messen, die sich nur implizit im Verhalten zeigen?

Stell dir vor, du müsstest Karten auf zwei Stapel verteilen, auf denen Spätzle und Hamburger sowie US-Amerikaner und Schwaben zu sehen sind. Wahrscheinlich würde es dir leichter fallen, Amerikaner mit Hamburgern und Spätzle mit Schwaben zusammen auf einen Stapel zu packen, als Spätzle mit Amerikanern und Hamburger mit Schwaben. Der IAT macht sich dieses Prinzip zunutze. Damit du den IAT besser verstehst, lassen wir dich zuerst einen Probe-IAT zu Hunden und Katzen machen. Aber bevor du damit anfängst, sind wir schon einmal gespannt, ob du deine Reaktionen vorhersagen kannst. Klicke weiter, um eine Vorhersage für einen Probe-IAT zu Hunden und Katzen zu machen.

Bitte klicke auf 'Weiter', um zur nächsten Seite mit der Übungsvorhersage zu gelangen.


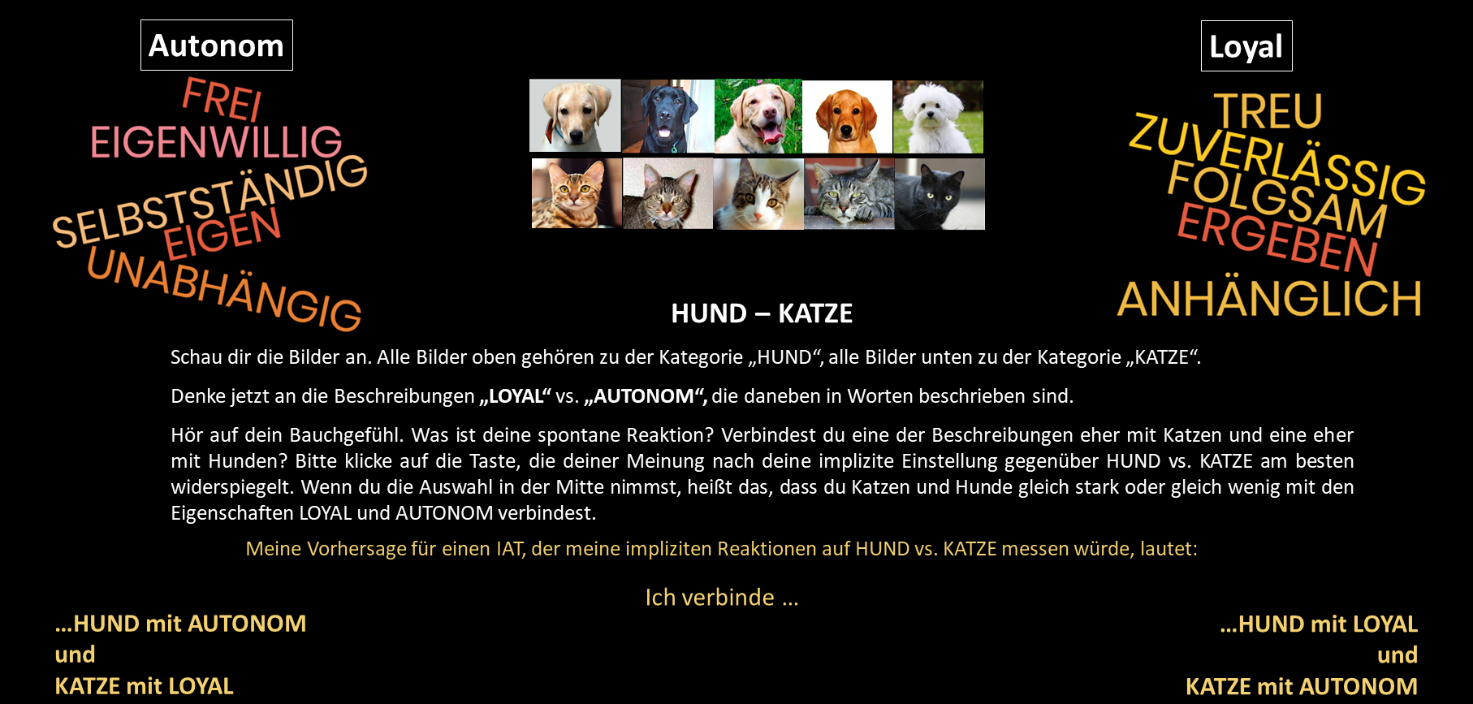


*loyal: treu, zuverlässig, folgsam, ergeben, anhänglich*

*autonom: frei, eigenwillig, selbstständig, eigen, unabhängig*

**Transition slide:**

WIE GENAU WAR DEINE VORHERSAGE?

Nun wirst du einen IAT – den impliziten Assoziationstest – bearbeiten. Der IAT misst deine impliziten Einstellungen zu Hunden und Katzen. Konntest du diese richtig vorhersagen?

Die Aufgabe geht so: Du sollst verschiedene Begriffe oder Bilder kategorisieren. Das heißt, bei jedem Durchgang erscheint ein Wort oder ein Bild in der Mitte des Bildschirms, welches du einer bestimmten Kategorie zuordnen sollst.

Beim IAT ist es wichtig, die Wörter und Bilder so SCHNELL wie möglich zu sortieren, aber dabei gleichzeitig so WENIG FEHLER wie möglich zu machen.

Wenn du zu langsam reagierst, oder zu viele Fehler machst, können wir dein Ergebnis nicht auswerten.

Es geht darum, eine von zwei Tasten zu drücken, wenn ein Wort oder ein Bild auf dem Bildschirm erscheint, und es damit einer Kategorie auf der linken oder rechten Seite zuzuordnen.

Die Kategorien, um die es in der jeweiligen Aufgabe geht, werden jeweils oben in der linken und rechten Ecke des Bildschirms zu sehen sein.

Drücke die Taste A, wenn etwas zur linken Seite gehört.

Drücke die Taste 5 (rechts, auf dem Ziffernblock), wenn etwas zur rechten Seite gehört.

Das Bild unten zeigt, welche Tasten wir genau meinen. Lass deine Finger während der Aufgabe am besten immer auf diesen Tasten liegen.

Ein rotes X erscheint jedes Mal, wenn du einen Fehler machst - korrigier den Fehler, indem du die andere Taste drückst.

(zum Fortfahren Leertaste drücken)

Jetzt kommt ein Probe-IAT. Du hast gerade eine Vorhersage gemacht,

wie deine spontanen Reaktionen zu Katzen bzw. Hunden sein werden.

Dieser IAT misst deine spontanen Reaktionen.

Die zwei Stimuli, zwischen denen du in diesem Block unterscheiden sollst, sind Fotos, die zu den Kategorien KATZE oder HUND gehören.

Drücke Taste A, wenn ein Bild zu der Kategorie HUND gehört.

Drücke Taste 5, wenn ein Bild zu der Kategorie KATZE gehört.

(zum Fortfahren Leertaste drücken)

Zur Erinnerung:

Behalte deine Finger während der Aufgabe durchgehend auf den Tasten 'A' und '5'.

Wenn du die falsche Taste drückst, erscheint ein rotes X auf dem Bildschirm.

Zum Korrigieren drücke die andere Taste.

*All instruction slides that follow are comparable in wording. The specific blocks are:*

*Participants now complete a short version of a dog-cat IAT with five blocks.*

*Block 1 has participants sort pictures of cats and dogs.*

*Block 2 has participants sort words pertaining to the concepts “loyal” and “autonomous”.*

*In Block 3, participants sort pictures of cats and dogs and words simultaneously.*

*In Block 4, participants sort pictures to reversed sides.*

*In Block 5, participants again sort pictures and words at the same time, but the pairing is reversed.*

*See stimuli in prediction slide above.*

**Transition slide 1 after the Cat-Dog IAT:**

DU HAST DEN TRAININGS-IAT GESCHAFFT.

Wie erging es dir dabei? Fielen dir manche Zuordnungen leichter als andere? Der IAT misst, ob es dir leichter fällt, die Kategorien autonom und Hunde gemeinsam zu sortieren oder autonom und Katzen. Daraus leiten wir deine spontanen Reaktionen ab. Bitte beschreibe deine Reaktion auf den Probe-IAT kurz in deinen eigenen Worten. Was wird dein Ergebnis zeigen?

*Large text box*

Continue [button]

Gleich wirst du weitere IATs mit Männern und Frauen als Kategorie machen. Nun, da du die Aufgaben kennst, was denkst du wie deine spontanen Reaktionen sein werden?

(zum Fortfahren Leertaste drücken)

**Transition slide 2 after the Cat-Dog IAT:**

Bitte denke über deine IMPLIZITEN EINSTELLUNGEN zu Frauen und Männern nach.

Es geht um diese Charakterisierungen von Männern und Frauen:

KARRIERE und FAMILIE

EMOTIONAL und RATIONAL

MATEHMATIK und SPRACHE

GEMEINWOHL und EIGENWOHL

SPORT und KÜNSTE

Was denkst du, wie deine Ergebnisse in einem Impliziten Assoziationstest (IAT) aussehen würden? Wird er zeigen, dass für dich einzelne Begriffe spontan eher zu Frauen oder eher zu Männern passen? Es geht um deine spontanen Reaktionen, achte also auf dein erstes Gefühl beim Blick auf die Bilder und die Worte!

Kennst du deinen spontanen Reaktionen, Bilder und Assoziationen? Nur du kannst uns das sagen, also versuch dein Bestes!

Nach deinen Einschätzungen wirst du die IATs tatsächlich durchführen. Wie gut kannst du deine Ergebnisse vorhersagen?

____________________________________________________________________

**Slides 3-8 (presented in individually randomized orders)***Whether men or women were on top was randomized between participants, but left constant for each individual participant. The sides of the concepts were also counter-balanced between participants (see paper).*


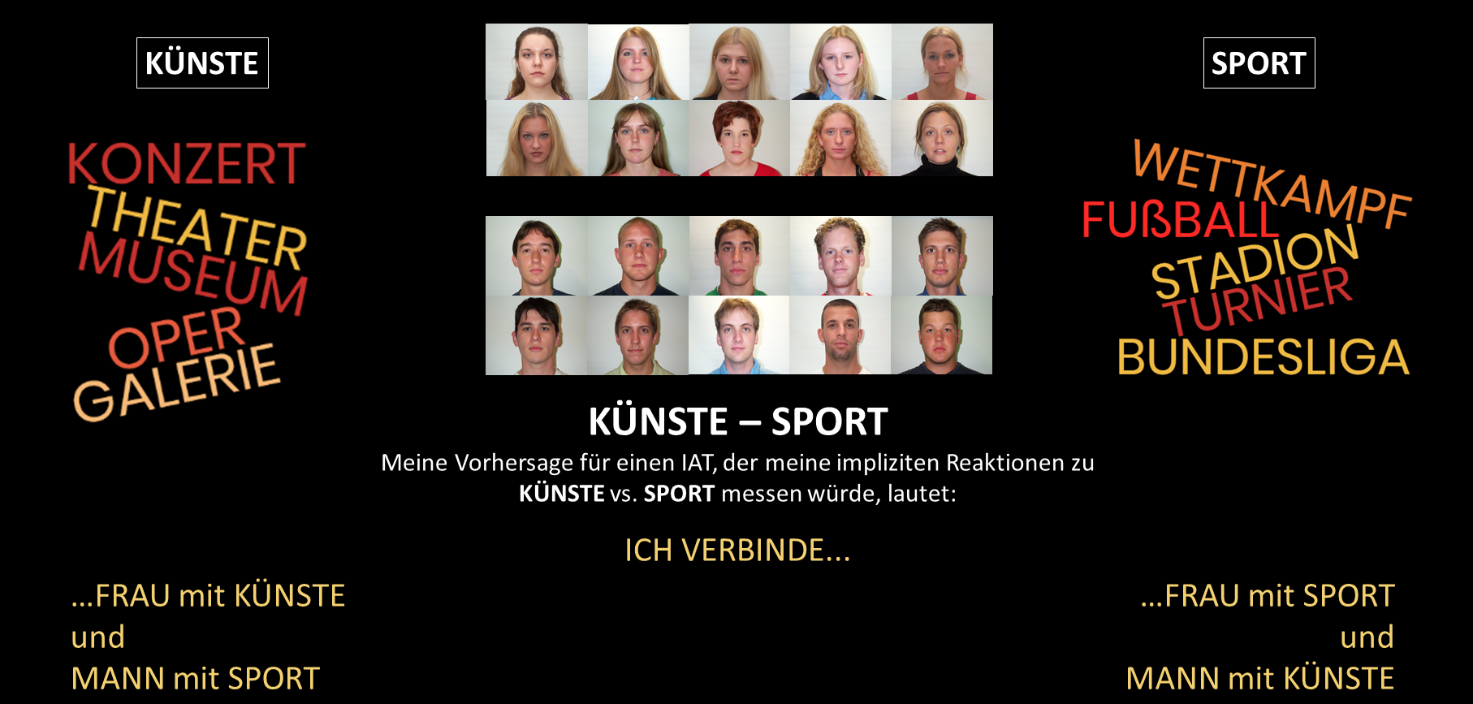


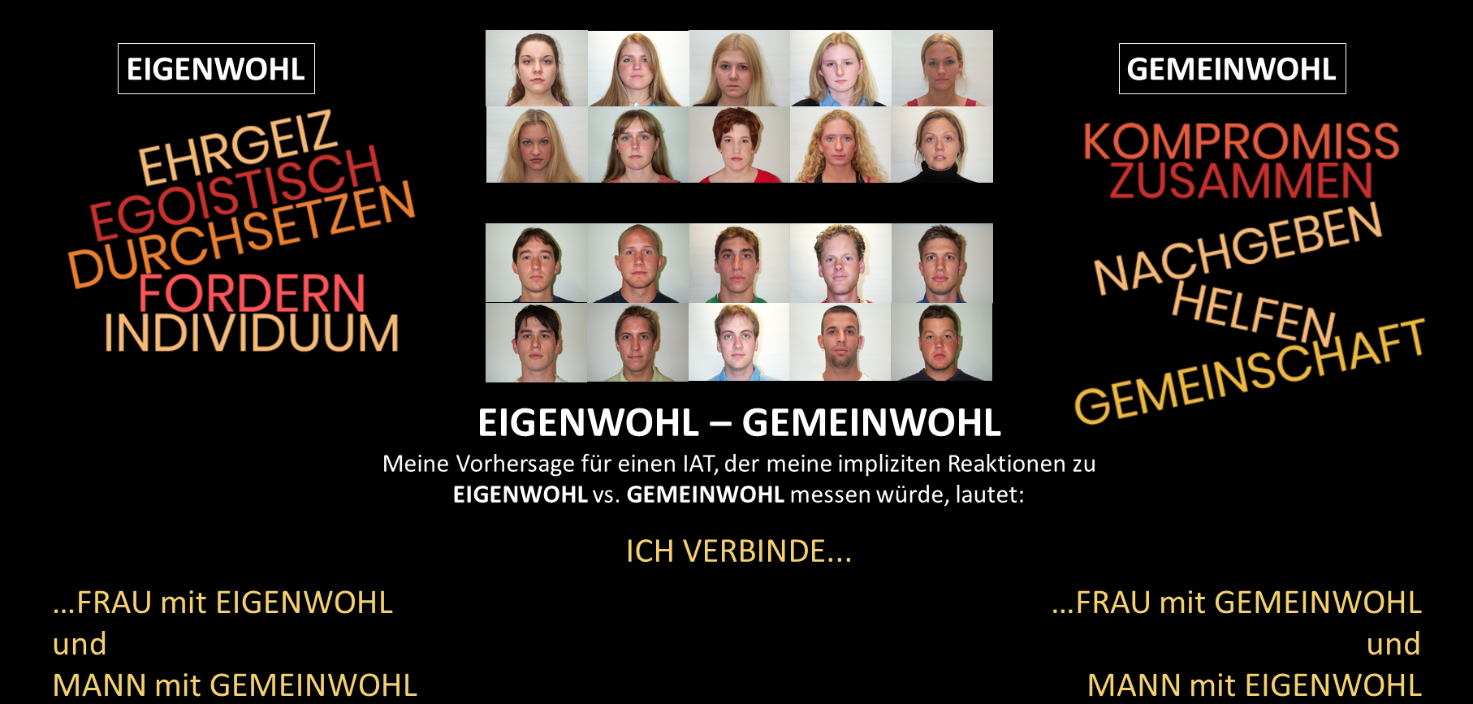


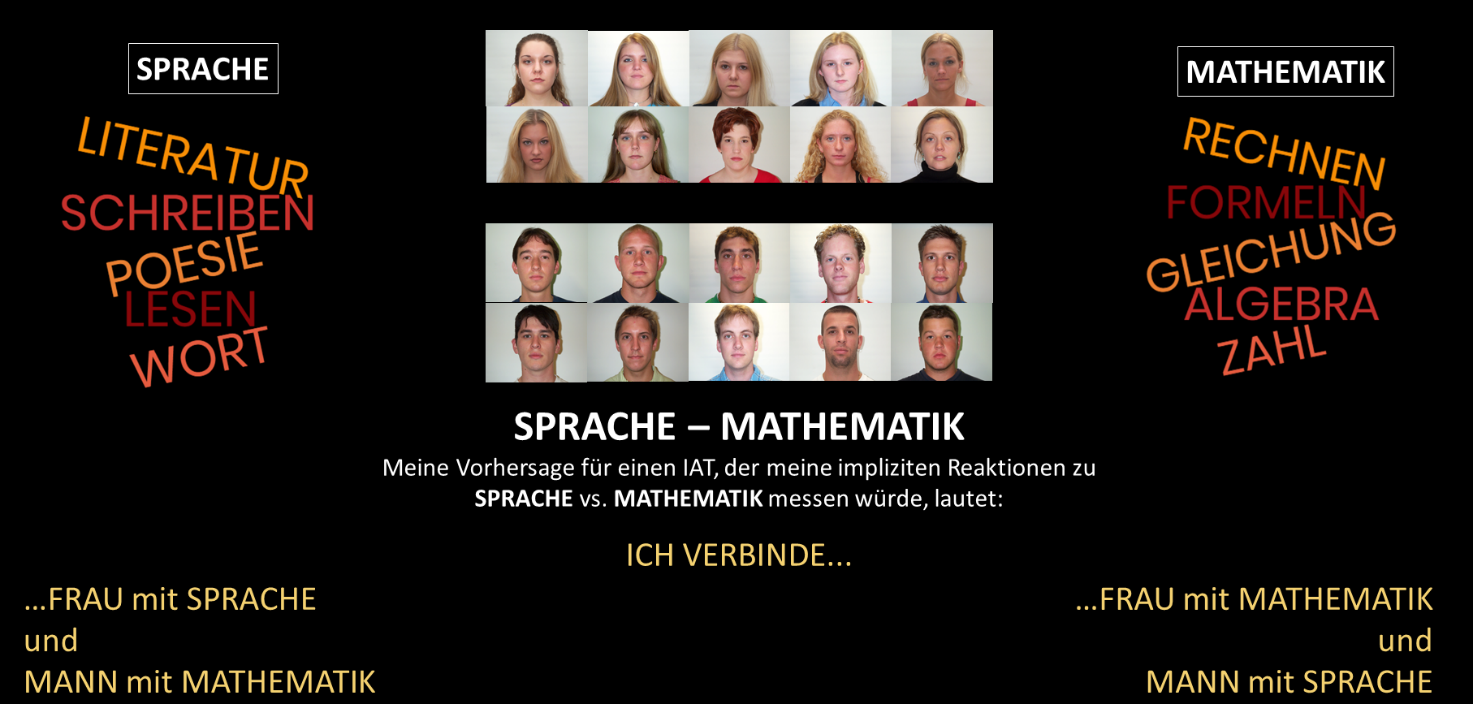


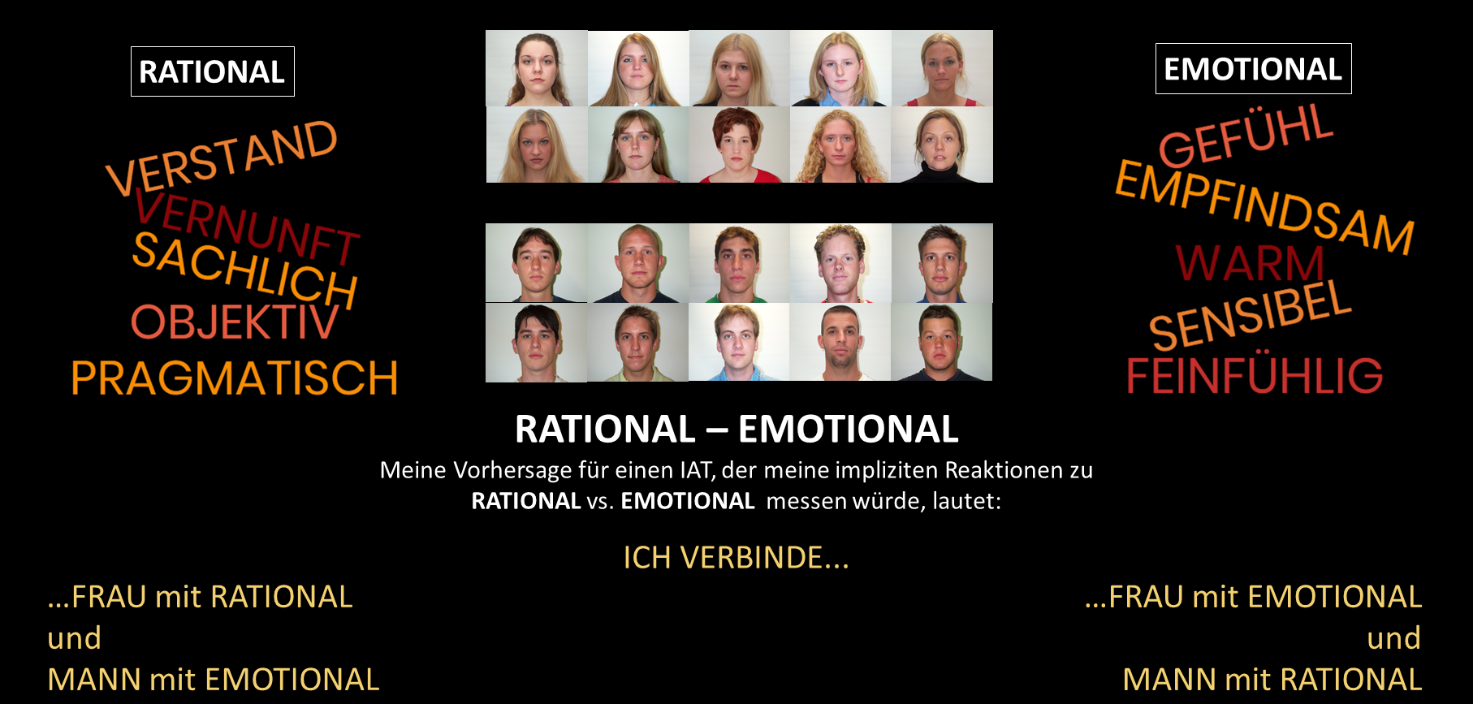


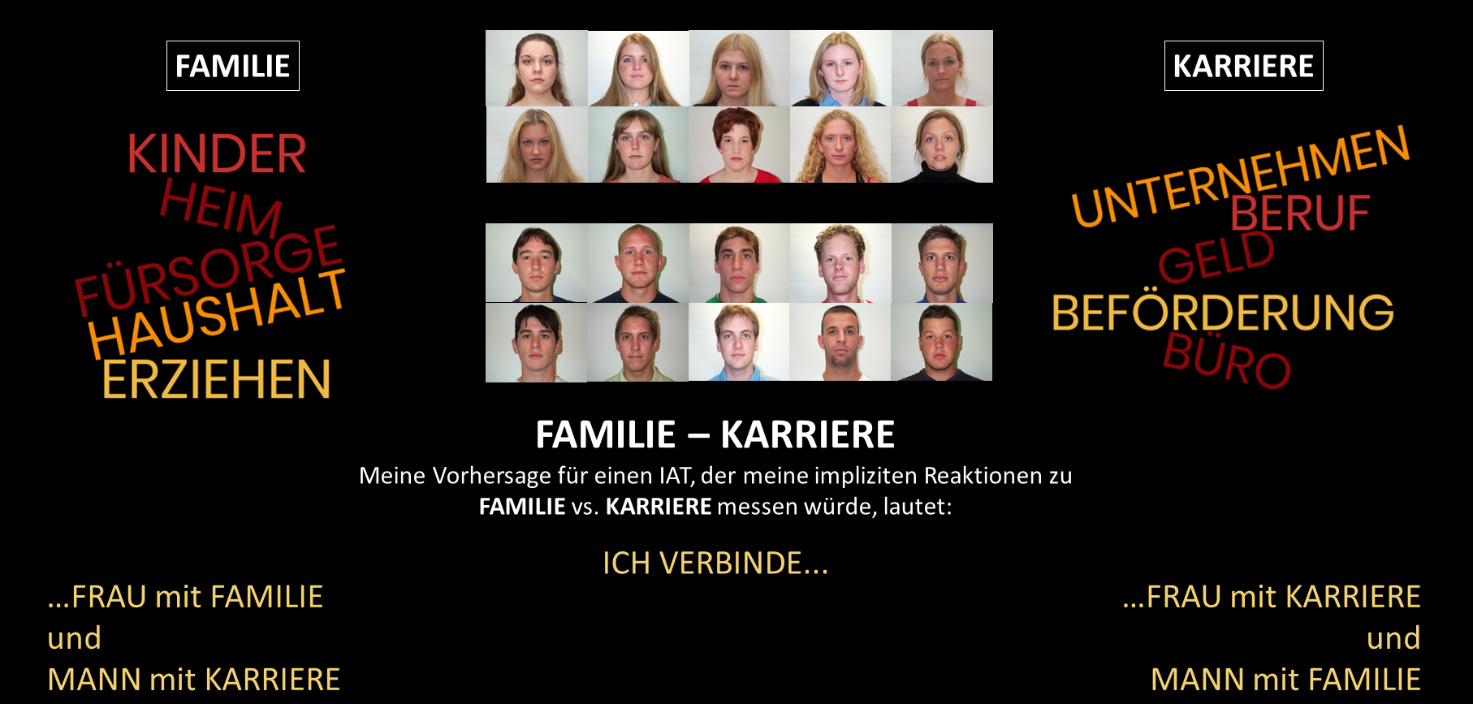


Nun beginnen die IATs. Das Prinzip ist wie zuvor, als du Hunde- und Katzenbilder sortiert hast.

Zur Erinnerung:

Bitte mache möglichst KEINE FEHLER und reagiere so SCHNELL wie möglich.

Behalte deine Finger während der Aufgabe durchgehend auf den Tasten 'A' und '5'*.*

***Participants now completed the five IATs, in individually randomized orders. Instructions were similar to the sample instructions shown above for the dog-cat IAT.***

**Post-IAT explicit attitudes slide:**

WIE SIND MÄNNER UND FRAUEN?

Zum Abschluss fragen wir dich noch mal nach deinen Einstellungen zu Männern und Frauen mit den Skalen, die du am Anfang schon benutzt hast.

Uns interessiert, ob sich deine Einstellungen verändert hat oder gleich geblieben sind.

Weiter geht es mit der Leertaste.

*Wording of the post-experimental explicit ratings were the same as in the beginning.*

*Demographics*

1. Alter ________________
2. Geschlecht: (x) weiblich (x) männlich (x) andere
3. Migrationshintergrund:
   - - 1. Ich habe keinen Migrationshintergrund
       2. In Deutschland geboren, aber einer meiner Eltern war bei seiner/ihrer Geburt nicht deutsch
       3. In Deutschland geboren, aber meine beiden Eltern waren bei ihrer Geburt nicht Deutsch.
       4. Nicht in Deutschland geboren, im Laufe meines Lebens nach Deutschland gekommen
4. [if C4 was selected] In welchem Alter bist du nach Deutschland gezogen?
5. Welche Staatsbürgerschaft hast du? (x) Deutsch (x) Deutsch und andere (x) nur andere
6. [If E3 was selected] Welche Staatsbürgerschaft hast du? ___________________________
7. Mit welcher Sprache bist du aufgewachsen? (x) nur Deutsch (x) Deutsch und andere Sprache (x) kein Deutsch
8. [if not G1] Welche andere Sprache(n) hast du zu Hause gesprochen? ________________________________
9. Wie ist dein Status an der Universität zu Köln? (x) studiere im Bachelor (x) studiere im Master (x) kein Student, aber studiert (x) kein Student, nie studiert
10. [if I1 or I2 was selected] In welchem Semester bist du? ____
11. Was ist dein Studienfach? (x) Psychologie im Hauptfach (x) ein anderer Studiengang (nicht Psychologie im Hauptfach) mit Kursen in Psychologie (x) ein anderer Studiengang (nicht Psychologie im Hauptfach) ohne Kurse in Psychologie (x) Ich studiere nicht und habe nie studiert
12. Was ist dein Studienfach? _____________________________________________
13. Hast du jemals etwas über den IAT oder ähnliche implizite Messmethoden gehört? (x) ja (x) nein
14. Wenn ja, wo? _____________________________________________________
15. Hast du, bevor du heute an dieser Studie teilgenommen hast, schon einmal hier oder auch woanders (z. B. online) einen IAT gemacht? (x) ja (x) nein
16. Wenn ja, beschreibe deine Erinnerung: ___________________________________
17. Hast du den Eindruck, schonmal an einer ähnlichen Studie teilgenommen zu haben? (x) ja (x) nein
18. Ich habe bereits an einer Studie teilgenommen, bei der ich meine IAT-Werte für ... angeben musste.
    1. Sozial und ethnisch
    2. Backwaren
    3. Berufsgruppen
    4. Einstellungen zu Geschlechtern
19. Kannst du deine Strategie für die IAT Vorhersage mit deinen eigenen Worten beschreiben? [large text box] __________________________________
20. Wie sehr hast du bei der Vorhersage deiner IAT-Werte auf dein Bauchgefühl geachtet?

Überhaupt nicht (o) (o) (o) (o) (o) (o) (o) Sehr stark

1. Wie sehr hast du bei der Vorhersage deiner IAT-Werte darauf geachtet, was du gesellschaftlich gelernt hast?

Überhaupt nicht (o) (o) (o) (o) (o) (o) (o) Sehr stark

1. Hast du noch Feedback für uns? [large text box] __________________________________
